# A cycle-consistent adversarial network for brain PET partial volume correction without prior anatomical information

**DOI:** 10.1007/s00259-023-06152-0

**Published:** 2023-02-20

**Authors:** Amirhossein Sanaat, Hossein Shooli, Andrew Stephen Böhringer, Maryam Sadeghi, Isaac Shiri, Yazdan Salimi, Nathalie Ginovart, Valentina Garibotto, Hossein Arabi, Habib Zaidi

**Affiliations:** 1 Division of Nuclear Medicine and Molecular Imaging, Geneva University Hospital, CH-1211 Geneva, Switzerland; 2 Persian Gulf Nuclear Medicine Research Center, Department of Molecular Imaging and Radionuclide Therapy (MIRT), Bushehr Medical University Hospital, Faculty of Medicine, Bushehr University of Medical Sciences, Bushehr, Iran; 3 Department of Medical Statistics, Informatics and Health Economics, Medical University of Innsbruck, Schoepfstr. 41, Innsbruck, Austria; 4 Geneva University Neurocenter, University of Geneva, Geneva, Switzerland; 5 Department of Psychiatry, Geneva University, Geneva, Switzerland; 6 Department of Basic Neuroscience, Geneva University, Geneva, Switzerland; 7 Department of Nuclear Medicine and Molecular Imaging, University of Groningen, Groningen, Netherlands; 8 Department of Nuclear Medicine, University of Southern Denmark, Odense, Denmark

**Keywords:** PET, Brain, Partial volume effect, Partial volume correction, Deep learning

## Abstract

**Purpose:**

Partial volume effect (PVE) is a consequence of the limited spatial resolution of PET scanners. PVE can cause the intensity values of a particular voxel to be underestimated or overestimated due to the effect of surrounding tracer uptake. We propose a novel partial volume correction (PVC) technique to overcome the adverse effects of PVE on PET images.

**Methods:**

Two hundred and twelve clinical brain PET scans, including 50 ^18^F-Fluorodeoxyglucose (^18^F-FDG), 50 ^18^F-Flortaucipir, 36 ^18^F-Flutemetamol, and 76 ^18^F-FluoroDOPA, and their corresponding T1-weighted MR images were enrolled in this study. The Iterative Yang technique was used for PVC as a reference or surrogate of the ground truth for evaluation. A cycle-consistent adversarial network (CycleGAN) was trained to directly map non-PVC PET images to PVC PET images. Quantitative analysis using various metrics, including structural similarity index (SSIM), root mean squared error (RMSE), and peak signal-to-noise ratio (PSNR), was performed. Furthermore, voxel-wise and region-wise-based correlations of activity concentration between the predicted and reference images were evaluated through joint histogram and Bland and Altman analysis. In addition, radiomic analysis was performed by calculating 20 radiomic features within 83 brain regions. Finally, a voxel-wise two-sample t-test was used to compare the predicted PVC PET images with reference PVC images for each radiotracer.

**Results:**

The Bland and Altman analysis showed the largest and smallest variance for ^18^F-FDG (95% CI: − 0.29, + 0.33 SUV, mean = 0.02 SUV) and ^18^F-Flutemetamol (95% CI: − 0.26, + 0.24 SUV, mean =  − 0.01 SUV), respectively. The PSNR was lowest (29.64 ± 1.13 dB) for ^18^F-FDG and highest (36.01 ± 3.26 dB) for ^18^F-Flutemetamol. The smallest and largest SSIM were achieved for ^18^F-FDG (0.93 ± 0.01) and ^18^F-Flutemetamol (0.97 ± 0.01), respectively. The average relative error for the kurtosis radiomic feature was 3.32%, 9.39%, 4.17%, and 4.55%, while it was 4.74%, 8.80%, 7.27%, and 6.81% for NGLDM_contrast feature for ^18^F-Flutemetamol, ^18^F-FluoroDOPA, ^18^F-FDG, and ^18^F-Flortaucipir, respectively.

**Conclusion:**

An end-to-end CycleGAN PVC method was developed and evaluated. Our model generates PVC images from the original non-PVC PET images without requiring additional anatomical information, such as MRI or CT. Our model eliminates the need for accurate registration or segmentation or PET scanner system response characterization. In addition, no assumptions regarding anatomical structure size, homogeneity, boundary, or background level are required.

**Supplementary Information:**

The online version contains supplementary material available at 10.1007/s00259-023-06152-0.

## Introduction

Over the recent decades, positron emission tomography (PET) imaging, among other molecular imaging modalities, has gained importance in preclinical, clinical, and research fields. PET is widely used in the assessment of oncology patients, cardiac pathologies, and various neurological disorders, including Alzheimer’s Disease (AD), Parkinson’s Disease (PD), and epilepsy. PET provides functional information useful in the assessment of a variety of metabolic processes, such as tissue metabolism, protein accumulation, and neurotransmission pathways [1, 2]. Accurate and reliable quantification is a major strength of molecular PET imaging as it allows us to accurately assess molecular pathways and various diseases in their earliest phases. For instance, accurate localization and/or quantification of tracer uptake in malignant lesions is the basis for pre- and post-treatment evaluations in neurooncology. In addition, accurate delineation of tumor contours is crucial in monitoring treatment response and radiation therapy planning.

The limited spatial resolution and low signal-to-noise ratio are the main drawbacks of PET imaging, making accurate quantitative analysis a challenging task in clinical practice. The partial volume effect (PVE) results from the poor spatial resolution of PET scanners, typically in the range of 3.5 to 6 mm full-width-half-maximum (FWHM). As a result of PVE, the intensity of a particular voxel is affected not only by the tracer concentration of the tissue in which the voxel is located but also by the surrounding tissues/organs. In addition, the physical size and shape of the volume of interest (VOI) and its contrast relative to surrounding regions affect PVE. Therefore, correction for PVE is mandatory for reliable quantitative measurements of physiological parameters and image-derived metrics, such as the standardized uptake value (SUV) or tumor-to-background ratio (TBR) for specific VOIs. This is particularly relevant when the pathology itself affects the volume of the target regions, as is the case in neurodegenerative diseases which are typically associated with atrophy.

Partial volume correction (PVC) techniques can overcome the adverse effects of PVE on PET images. Studies have shown that PVC improves diagnostic accuracy and SUV quantification [3], estimation of tracer uptake in plaque in large vessels or in an atrophied gray matter [4], and measurement of ventricular mass [5], in addition to improving overall image quality for ^18^F-Flortaucipir and amyloid PET tracers [6, 7]. Moreover, PVC PET images allow for the quantification of different physiologic processes in the brain, including cerebral blood flow, glucose metabolism, neuroreceptor binding, and tumor metabolism [8]. Applying PVC methods also proved to improve the statistical power in cross-sectional [9] and longitudinal [6] analyses in quantitative amyloid imaging. PVC can also eliminate confounding results in studies of aging [10] or atrophy effects in the brain [11, 12]. For instance, PVC prevents the underestimation of physiologic measurements due to the loss of cerebral volume resulting from healthy aging processes. A number of studies demonstrated that PVC improves clinical classification performance in AD [13] and PD [14] research. It can be concluded that PVC is necessary to ensure that measurements are truly quantitative for different regions within the brain. To this end, a number of PVC techniques have been developed and implemented with varying degrees of success [15–17].

Most popular PVC methods for brain PET imaging, such as Meltzer’s method [15], Müller-Gärtner (MG) [16], or the geometric transfer matrix (GTM) method [17], typically require other imaging modalities, such as CT or MRI as a priori anatomical information. This dependence gives rise to a key drawback, namely the need for accurate co-registration of PET to CT or MR images. This dependency means that misregistration or inaccurate segmentation contributes to errors in PVC. Other methods use the PET scanner’s point spread function (PSF). The downside of these methods is that they require an accurate estimate of the spatially varying PSF, which might be difficult to measure [17]. Other methods require dedicated reconstruction software, which is readily not available for all PET/CT or PET/MRI systems. The mentioned downfalls of the current PVC methods highlight an unmet need for an end-to-end method to produce high-resolution PET images without the need for additional anatomical images and prior knowledge of PET scanner characteristics, tumor and VOI size, shape, or background level. Lu et al. assessed the impact of Müller-Gärtner (MG) and iterative Yang (IY) PVC on ^11^C-UCB-J brain PET images for finding synaptic vesicle glycoprotein 2A (SV2A), which has been suggested as an indicator of synaptic density in Alzheimer’s disease (AD) [18]. Onoue et al. compared CT and MRI-based PVC in brain ^18^F-FDG PET and discussed the advantages of PVC using CT images [19]. An error propagation analysis was also performed for seven PVC methods by Oyama et al., where they showed around 30% bias in small and thin regions in AD patients with and without PVC [20].

Recently, machine learning (ML), especially deep learning (DL) as a subset of ML, has been increasingly used in various applications of PET imaging [21–23]. With advances in both DL algorithms and computational power, a paradigm shift favoring DL-based PVC approaches might be very promising toward the development of accurate and robust methods.

This work proposes a novel anatomical imaging-free DL-assisted PVC algorithm and evaluates its performance using clinical brain studies acquired with four PET neuroimaging radiotracers. The method is an end-to-end PVC pipeline, which inputs a low-resolution brain PET image to generate a high-quality PVC image, which does not require anatomical imaging and a priori knowledge of the PSF, VOI size, shape, or background level.

## Materials and methods

### PET/CT and MRI data acquisition

Patients undergoing a brain PET/CT/MRI scan collected between April 2017 and February 2020 at Geneva University Hospital were enrolled in this study. The study protocol was approved by the institution’s ethics committee, and all patients gave written informed content. The two hundred and twelve patients dataset were acquired following injection of four different PET neuroimaging radiotracers (50 ^18^F-FDG, 50 ^18^F-Flortaucipir, 36 ^18^F-Flutemetamol, and 76 ^18^F-FluoroDOPA). The corresponding CT and T1-weighted MR images were also used in this study. A combination of healthy patients and those diagnosed with different pathologies, such as neurodegenerative disease, cannabis use disorder, and internet gaming disorder, were considered for training the model to increase the generalizability of our method. The corresponding demographic details are summarized in Table 1.

**Table 1:** Demographics of the patient population included in this study protocol

	Dataset (5 cross-validation)	Scanning protocol
^18^F-FDG		
Number	100 (50 actual + 50 LB)	Delay between injection and scanning: 32 ± 6 min Injected activity: 208 ± 14 MBq Scan duration: 20 min
Male/female	38/12	
Age (mean ± SD)	69 ± 5	
Indication/diagnosis	Cognitive symptoms of possible neurodegenerative etiology	
^18^F-FluoroDOPA		
Number	100 (50 actual + 50 LB)	Delay between injection and scanning: 0 min Injected activity: 185 ± 12 MBq Scan duration: 90 min
Male/female	31/19	
Age (mean ± SD)	25 ± 4	
Indication/diagnosis	Healthy patients and patients with a psychiatric disorder	
^18^F-Flortaucipir		
Number	100 (36 actual + 64 LB)	Delay between injection and scanning: 76 ± 8 min Injected activity: 205 ± 10 MBq Scan duration: 30 min
Male/Female	20/16	
Age (mean ± SD)	53 ± 9	
Indication/diagnosis	Cognitive symptoms of possible neurodegenerative etiology	
^18^F-Flutemetamol		
Number	100 (76 actual + 24 LB)	Delay between injection and scanning: 91 ± 6 min Injected activity: 199 ± 11 MBq Scan duration: 20 min
Male/female	42/28	
Age (mean ± SD)	66 ± 8	
Indication/diagnosis	Cognitive symptoms of possible neurodegenerative etiology	

Data augmentation using the Laplacian blending (LB) technique was used to increase the sample size

Attenuation and scatter-corrected PET images as well as T1-weighted MR images were acquired on the Biograph mCT scanner and 3 T MAGNETOM Skyra scanner (Siemens Healthcare, Erlangen, Germany), respectively. The PET scanning protocol for the different radiotracers, including injected activities, scan time durations, and delay times between injection and PET scanning, is summarized in Table 1. MRI data acquisition protocol was similar for the various radiotracers. The PET/CT/MRI scanning protocol details were summarized in Supplementary Table 1.

### Data processing and image registration

After cropping PET and MR images, they were coregistered to the corresponding standard brain template defined into Montreal Neurological Institute (MNI) (Montreal Neurological Institute, McGill University) standard stereotactic space [24] using the 3D Slicer software [25]. An affine registration method with 12 degrees of freedom was employed for all images [26]. Because PET and CT images acquired on the PET/CT scanner were already registered, PET images were registered to the MNI template, and the resulting registration matrix was applied to CT images. Subsequently, T1-weighted MRI was registered to CT images. All images were visually assessed to ensure accurate registration between PET, CT, and MR images.

### Data augmentation

Since the number of cases for each radiotracer was not similar, the effect of dataset size on model performance was minimized using a previously developed augmentation method using the Laplacian blending (LB) technique, referred to as Robust-Deep [27], to increase the dataset size to a fixed number of 100 per radiotracer. The Robust-Deep technique increases the number of brain images by combining images of two different cases through a predefined mask to create a semi-realistic image, which can significantly enhance the robustness of the deep learning models.

### Partial volume correction

The Iterative Yang (IY) technique [4] was selected from the PET-PVC toolbox [28] for PVC. Unlike region-based PVC methods, where the corrections are only valid for voxels within a selected region to provide regional mean values (e.g., GTM, MGM), a voxel-by-voxel correction is applied to the whole image in the IY method. As such, the PVC image ${f}_{PVC}^{itr}(x)$ is estimated from the multiplication of the uncorrected PET image $f(x)$ and the ratio of artificial PET images ${f}_{a}^{itr}(x)$ and a blurred/smoothed version of this image (achieved by convolving ${f}_{a}^{itr}(x)$ with the PSF of the PET scanner):

1 $${f}_{\mathrm{PVC}}^{\mathrm{itr}+1}(x)=f(x).\left[\frac{{f}_{a}^{\mathrm{itr}}\left(x\right)}{{f}_{a}^{\mathrm{itr}}\left(x\right)\otimes PSF}\right]$$

where the artificial PET images ${f}_{a}^{itr}(x)$ is renewed at each iteration by multiplying the average value of the artificial PET ${f}_{a}^{itr}(x)$ at *j-*th regions (${A}_{j,{ f}_{PVC}^{itr}(x)}$) and anatomical probability of *j-*th regions at location x ${P}_{j}\left(x\right),$ which is extracted from MR images:

2 $${f}_{a}^{\mathrm{itr}}\left(x\right)={\sum\limits }_{j=1}^{\#\mathrm{Regions}}\left[{A}_{j,{ f}_{\mathrm{PVC}}^{\mathrm{itr}}(x)}.{P}_{j}(x)\right]$$

We initially considered the first PVC PET images as equal to the uncorrected PET images:

3 $${f}_{\mathrm{PVC}}^{0}\left(x\right)= f(x)$$

Ten iterations were used for PVC in this work. The FWHM of the 3D Gaussian convolution kernel was set to 3.0 × 3.0 × 3.0 mm.

### Network architecture

**Fig. 1:**
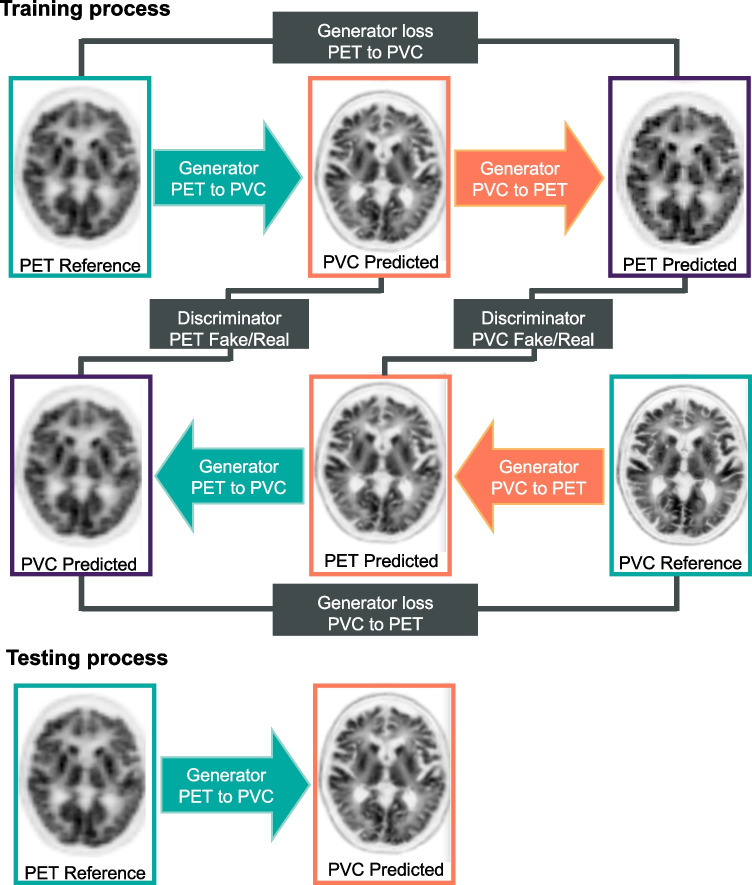
Schematic architecture of the cycle-consistent generative adversarial network (CycleGAN) model used for PET-PVC synthesis. The top panel depicts the training process, whereas the down panel shows the testing process

A Cycle-Consistent Generative Adversarial Network (CycleGAN), which learns a function to translate non-PVC PET images to PVC PET images (Fig. 1), was used in this work. The model consists of two GANs, including four main model architectures – two generators and two discriminators – as described in detail in Supplementary Table 2. The model training and evaluation were performed on an NVIDIA 2080Ti GPU with 11 GB memory running under Windows 10 operating system. We trained four different models with five-fold cross-validation for each radiotracer.

### Visual and quantitative evaluation for the test dataset

All images, namely original PVC and DL-predicted PVC images, were visually inspected to assess overall image quality and the presence of potential alterations and artifacts in tracer distribution.

Quantitative analysis was performed by calculating well-established metrics, such as structural similarity index metrics (SSIM), root mean squared error (RMSE), and peak signal-to-noise ratio (PSNR), showing geometric similarity between the DL-predicted and ground truth images, the level of error/noise, and the strength of the signal-to-noise ratio, respectively. Voxel-wise and region-wise activity concentration correlations between the DL-predicted and reference PET images were evaluated through joint histogram and Bland and Altman analysis. For region-wise analysis, 20 radiomic features from 83 brain regions were extracted through registering the reference and predicted images to the Hammers N30R83 brain atlas [29].

### Radiomics analysis

The image biomarker standardization initiative (IBSI) [30] compliant LIFEx software [31] was used for the extraction of the radiomic features. The list of the extracted radiomic features and their related categories are presented in Table 2. The relative bias between radiomic features extracted from the reference and DL-predicted PVC PET images were calculated over all radiotracers.

**Table 2:** Summary of the 20 radiomic features belonging to the 7 main categories calculated for the 83 brain regions

Radiomic feature category	Radiomic feature names
Conventional indices	SUV_mean_ SUV_std_ SUV_max_ SUV Q1 SUV Q2 SUV Q3 TLG (mL)
First-order features – histogram	Kurtosis Entropy_log10 Entropy_log2 Uniformity
Area under the curve of cumulative SUV histogram	AUC_CSH
Gray-level co-occurrence matrix (GLCM)	Homogeneity Energy Dissimilarity
Gray-level run length matrix (GLRLM)	Run percentage (RP)
Neighborhood gray-level difference matrix (NGLDM)	Contrast
Gray-level zone length matrix (GLZLM)	Short-zone emphasis (SZE) Low gray-level zone emphasis (LGZE) High gray-level zone emphasis (HGZE)

### Voxel-based statistical analysis

All T1-weighted, original non-PVC PVC, and DL-predicted PVC images for all PET neuroimaging tracers were pre-processed using FSL (FMRIB Software Library v6.0.1, Analysis Group, FMRIB, Oxford, UK). In each step, we initially pre-processed T1-weighted images and then applied transformation matrices to the original and DL-predicted PVC images. Therefore, the original non-PVC PVC and DL-predicted PVC PET images were identically pre-processed for each patient.

First, brain tissue was extracted from T1-weighted images using the BET function implemented within FSL (Brain Extraction Tool, FSL). Subsequently, skull-stripped T1-weighted images were used as a mask to extract brain tissue both from the original non-PVC PVC and DL-predicted PVC PET images for each patient. Afterward, T1-weighted images were registered to MNI standard space using the FLIRT function (FMRIB’s Linear Image Registration Tool, FSL). Then, the original non-PVC PVC and DL-predicted PVC PET images of each patient were registered to MNI space via FLIRT using the same transformation matrix employed for registering the T1-weighted image of that subject. We applied a linear image registration method that does not change the voxels’ values without smoothing to minimize the effect of pre-processing on the results. In each step, the outcome of pre-processing procedures was manually checked for potential errors, and appropriate corrections were performed when needed.

After these pre-processing steps, a mass univariate methodology of Statistical Parametric Mapping (SPM12; Welcome Centre for Human Neuroimaging, UCL, UK) was used to perform a voxel-wise two-sample t-test that compared the DL-predicted PVC with reference PVC PET images for each tracer dataset [32]. This analysis identifies voxel clusters with statistically significant differences in the DL-predicted PVC images compared to the reference PVC PET images. Statistical significance was determined at a voxel-wise threshold of *p* < 0.05 (family-wise error corrected), and no voxel clusters exceeding the threshold were determined.

## Results

**Fig. 2:**
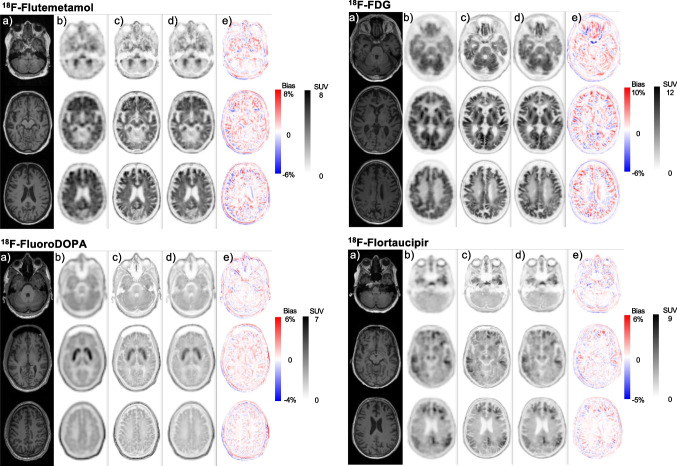
Representative slices of multi-tracer brain PET images of various patients showing (**a**) coregistered T1-weighted MRI, (**b**) non-PVC PET images, (**c**) reference corresponding MRI-guided PVC PET images, (**d**) DL-predicted PVC PET images, and (**e**) the corresponding bias maps

**Fig. 3:**
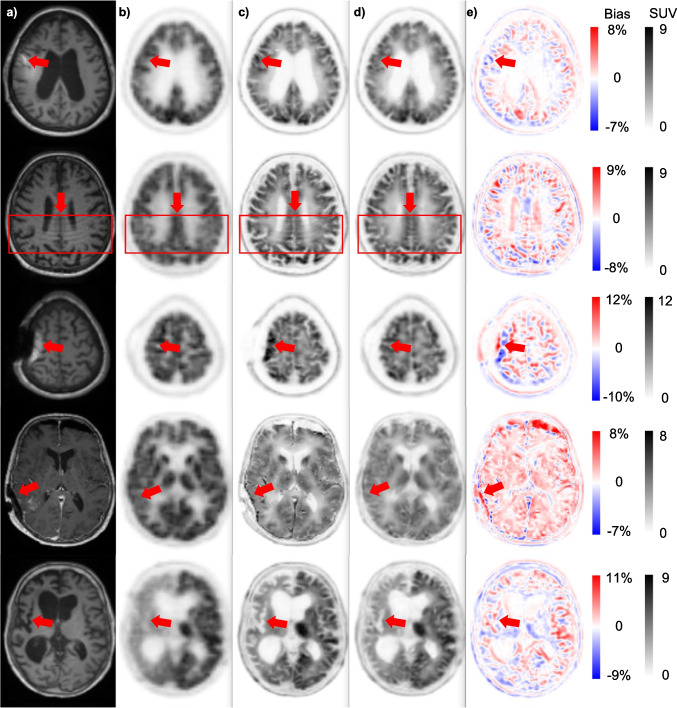
Five special cases of patients presenting with artifacts in MR images (first and second row), anatomical abnormalities or existence of external objects (third and fourth row), and ununiform activity distribution in PET images (last row): (**a**) coregistered T1-weighted MRI, (**b**) non-PVC PET images, (**c**) reference corresponding MRI-guided PVC PET images, (**d**) DL-predicted PVC PET images, and (**e**) the corresponding bias maps

All DL-predicted PVC PET images were considered visually adequate and comparable to the corresponding original PVC PET images, as exemplified in Figs. 2 and 3. In particular, Fig. 2 illustrates three different transaxial slices of MRI, non-PVC PET, reference MRI-based PVC PET, and the DL-predicted PVC PET images as well as the corresponding bias maps for the four different patients/radiotracers. The effectiveness of our model in terms of highlighting and enhancing the contours of the anatomical information in the DL-predicted PVC PET images is observable. It is worth noting that the DL-predicted PVC PET images are synthesized from only PET images as opposed to reference PVC PET which is generated from both MR and PET images. Figure 3 presents four abnormal cases depicting some artifacts and anatomical information loss in MR images, likely because of probable patient motion and the existence of metallic objects, such as a dental crown or a ventriculoperitoneal shunt or post-operative changes, causing artifacts in MR images. The reference PVC PET generated from MR and PET images highlights the propagation of MR artifacts/abnormalities into PVC PET images, while the DL-based PVC images are immune to these artifacts.

**Fig. 4:**
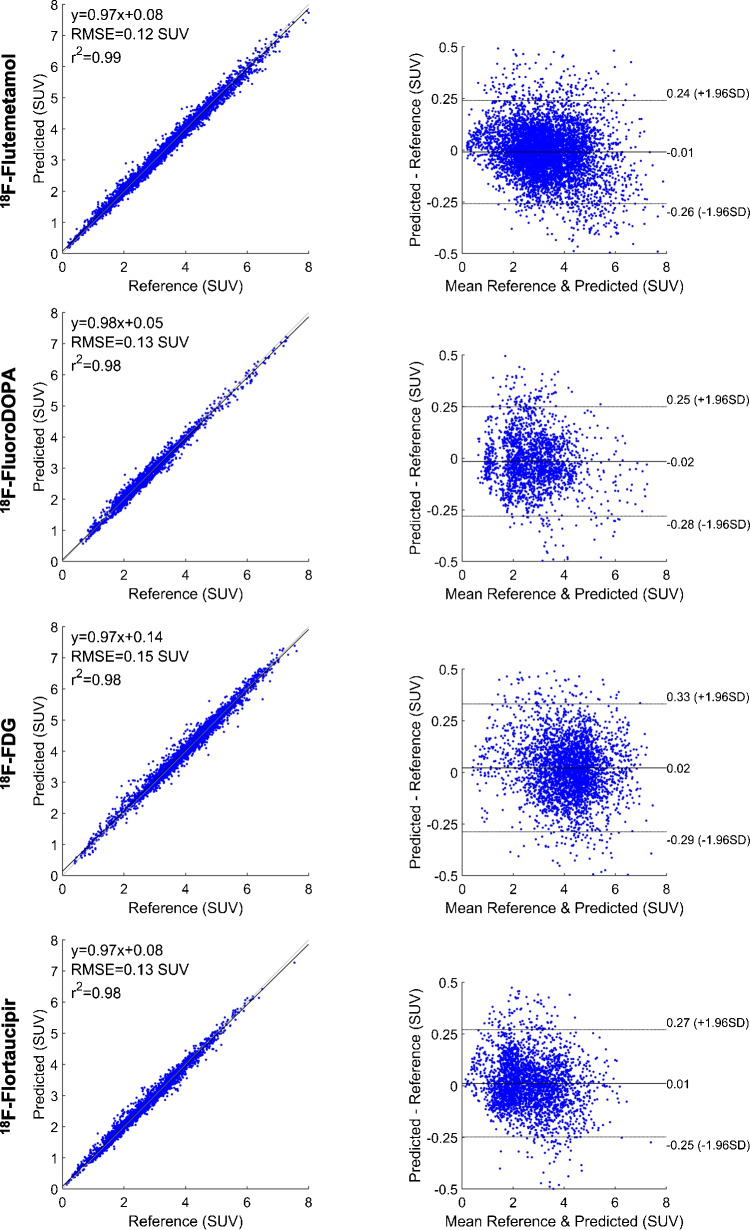
The Bland–Altman plots (right panel) and scatter plots (left panel) of SUV_mean_ differences in the 83 brain regions for various tracers. In the Bland–Altman plots, the black solid and dashed lines denote the mean and 95% confidence interval (CI) of the SUV differences, respectively. In the scatter plots, the black solid and dashed lines denote the linear regression line and identity line, respectively

The scatter and Bland and Altman plots for 83 brain regions over the test dataset for each radiotracer are illustrated in Fig. 4. For all radiotracers, the scatter plots show high correlations between SUVs calculated on DL-based PVC PET images and those on reference MRI-based PVC PET images, with a correlation coefficient (*R*^2^) larger than 0.98 and RMSE smaller than 0.15 SUV. The Bland and Altman plots show that the largest variance in terms of mean error and confidence interval (CI) was achieved for ^18^F-FDG (95% CI: − 0.29, + 0.33 SUV, mean = 0.02 SUV), whereas the smallest variance was obtained for ^18^F-Flutemetamol (95% CI: − 0.26, + 0.24 SUV, mean =  − 0.01 SUV).

**Fig. 5:**
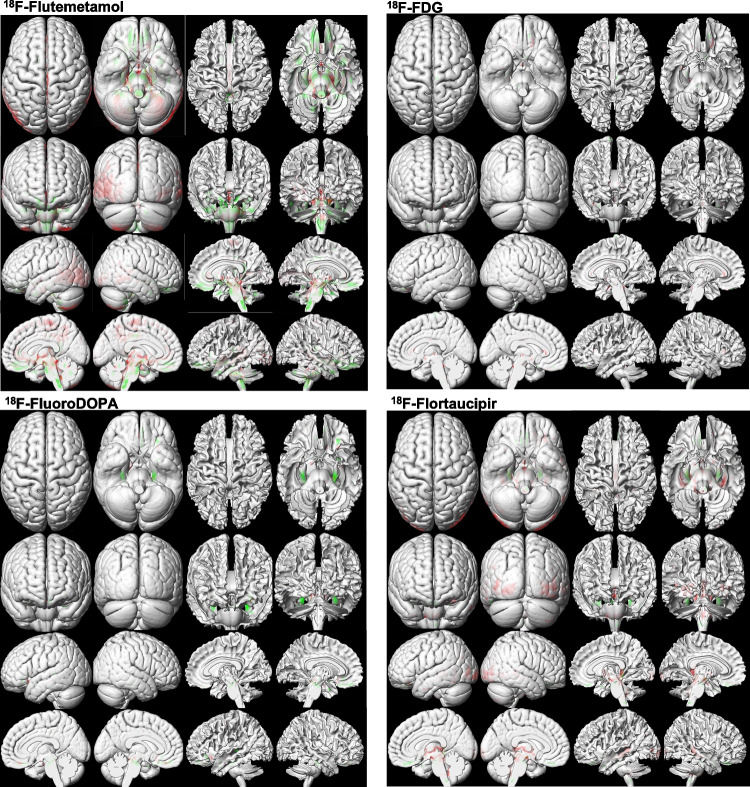
3D-rendered views of voxel-wise analysis of DL-predicted PVC images compared with reference PVC PET images for the different PET tracers. The images highlight voxel clusters with statistically significant differences compared with reference PVC PET images. In comparison to reference PVC images, the red regions represent voxels with statistically significant overestimation, while the green regions indicate voxels with statistically significant underestimation of tracer uptake

**Fig. 6:**
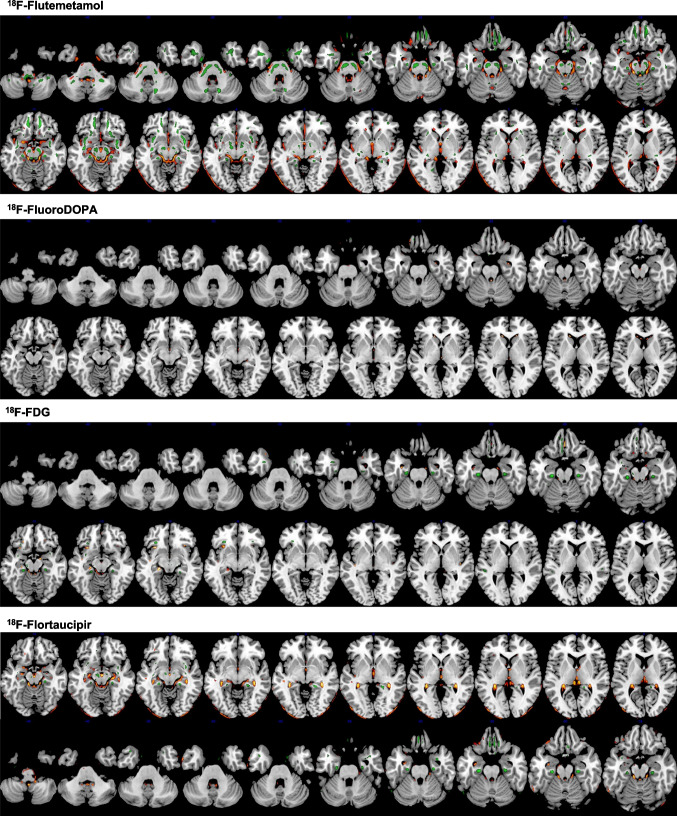
Multi-slice views of voxel-wise analysis of DL-predicted PVC PET images compared with reference PVC PET images for the different neuroimaging tracers. These images show voxel clusters with statistically significant differences in DL-predicted PVC PET images compared with reference PVC PET images at different slices of the brain. In comparison to reference PVC PET images, red/yellow regions represent voxel clusters with statistically significant overestimation, while green regions indicate voxel clusters with statistically significant underestimation of tracer uptake

Table 3 summarizes the outcome of quantitative evaluation metrics, including SSIM, PSNR, and RMSE for the different radiotracers. The PSNR varies from 29.64 ± 1.13 dB for ^18^F-FDG to 36.01 ± 3.26 dB for ^18^F-Flutemetamol. The smallest SSIM was achieved for ^18^F-FDG (0.93 ± 0.01), whereas the largest SSIM was obtained for ^18^F-Flutemetamol (0.97 ± 0.01). 3D-rendered views of voxel-wise statistical analysis of reference and DL-predicted PVC PET images for each PET tracer are shown in Fig. 5. The red and green regions represent voxels with statistically significant overestimation and underestimation of tracer uptake, respectively. In Fig. 6, clusters presenting with statistically significant differences between the DL-predicted and reference PVC PET images are depicted. By comparing the DL-based images with the original images, we have classified errors into two categories, namely overestimation and underestimation. The first describes the DL-predicted PVC PET voxels with a significantly lower value compared with the reference PVC PET voxels, while the latter describes voxels with a significantly higher value compared with the reference value. The DL model for ^18^F-FDG and ^18^F-FluoroDOPA datasets yielded a lower number of voxels with statistically significant differences compared with model performance in ^18^F-Flutemetamol and ^18^F-Flortaucipir datasets (Table 4).

**Table 3:** Comparison of the results obtained from the analysis of image quality in reference and DL-predicted PVC PET images for the different tracers in the test dataset

Tracer	SSIM	PSNR (dB)	RMSE
^18^F-FDG	0.93 ± 0.01	29.64 ± 1.13	6.16 × 10^-6^ ± 1.48 × 10^-6^
^18^F-Flortaucipir	0.97 ± 0.02	34.97 ± 1.97	2.31 × 10^-6^ ± 1.13 × 10^-6^
^18^F-Flutemetamol	0.97 ± 0.01	33.42 ± 1.92	2.82 × 10^-6^ ± 1.31 × 10^-6^
^18^F-FluoroDOPA	0.94 ± 0.02	36.01 ± 3.26	1.72 × 10^-6^ ± 1.55 × 10^-6^

*SSIM*, structural similarity index metrics; *PSNR*, peak signal-to-noise ratio; *RMSE*, root mean squared error

**Table 4:** Voxel-based statistical analysis between DL-predicted and original MRI-guided PVC for the different PET tracer

Tracer	Total number of voxels	Voxel number (%)	Mean T-value (± SD)
Underestimation^*^			
^18^F-Flortaucipir	1,827,095	1998 (0.10%)	6.11 (± 0.95)
^18^F-Flutemetamol	1,827,095	17,846 (0.97%)	6.33 (± 0.81)
^18^F-FDG	1,820,714	1730 (0.09%)	7.03 (± 1.39)
^18^F-FluoroDOPA	1,824,363	80 (0.00%)	6.27 (± 0.27)
Overestimation^**^			
^18^F-Flortaucipir	1,827,095	10,258 (0.56%)	6.09 (± 1.09)
^18^F-Flutemetamol	1,827,095	27,419 (1.50%)	6.49 (± 1.06)
^18^F-FDG	1,820,714	878 (0.04%)	6.31 (± 0.58)
^18^F-FluoroDOPA	1,824,363	400 (0.02%)	6.48 (± 0.40)

The models using ^18^F-FDG and ^18^F-FluoroDOPA images for predicting corresponding PVC images had fewer voxels with statistically significant differences, yielding better performance. Conversely, models using ^18^F-Flutemetamol and ^18^F-Flortaucipir images had more voxels with statistically significant differences, demonstrating worse prediction compared to ^18^F-FDG or ^18^F-FluoroDOPA. Here, “voxel number” represents the extent of a difference with statistical significance, and “T-values” represent the degree of a difference with statistical significance

*FEW*, family-wise error

*Voxels with significantly lower values (model underestimation); *p*-value (0.05, FWE corrected)

**Voxels with significantly higher values (model underestimation); *p*-value (0.05, FWE corrected)

**Fig. 7:**
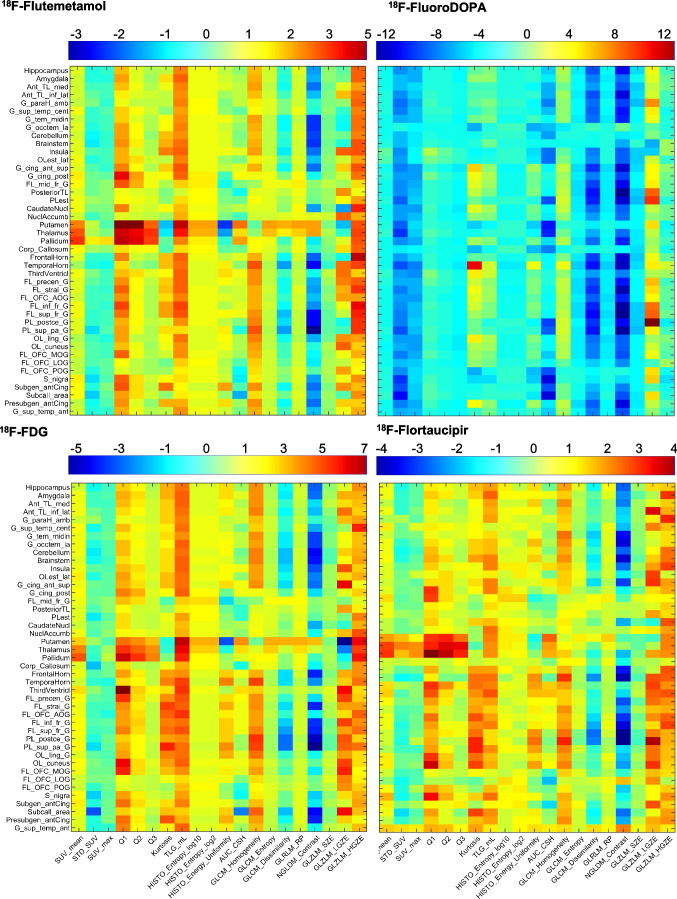
Heat maps of the relative error of 20 radiomics features calculated across 43 brain regions (for better presentation, the average of right and left regions were reported) for DL-predicted PVC PET images with respect to reference PVC PET images

The joint voxel-wise histogram analysis between reference and DL-predicted PVC PET images are depicted in Supplementary Fig. 1. The results are in good agreement with region-wise scatter plots. Figure 7 shows the relative error heat maps for 20 radiomic features and 83 regions for the different radiotracers. For a more concise presentation of the heat map, we reported the average of the left and right regions. The complete heat map for the 83 regions is depicted in Supplementary Figs. 2 and 3 to highlight abnormal cases where the left and right regions have different significantly different errors. The maximum underestimation and overestimation errors for each radiotracer can be appreciated from their corresponding color bar. It can be seen that the largest underestimation and overestimation is around 10% for ^18^F-FluoroDOPA. With this radiotracer, the SUV was mostly underestimated in the DL-predicted PVC PET images for all radiomic features, except gray-level zone length matrix low gray-level zone emphasis. The average relative error for the kurtosis radiomic feature was 3.32%, 9.39%, 4.17%, and 4.55%, whereas it was 4.74%, 8.80%, 7.27%, and 6.81% for NGLDM_contrast feature for ^18^F-Flutemetamol, ^18^F-FluoroDOPA, ^18^F-FDG, and ^18^F-Flortaucipir, respectively. The average relative error of HISTO_energy_Uniformity, a feature depicting the strength of the signal, varied from 2.81%, 5.93%, 4.30%, and 3.93% for ^18^F-Flutemetamol, ^18^F-FluoroDOPA, ^18^F-FDG, and ^18^F-Flortaucipir, respectively.

## Discussion

There is a growing interest in applying PVC for PET image interpretation and for quantifying various physiological parameters of interest in clinical and research settings. A variety of PVC algorithms have been developed; however, they are not yet widely applied in the clinical setting. One possible explanation for this fact could be that most available algorithms rely on certain assumptions that introduce uncertainty in the computation and ensuing quantification and require extra-anatomical images, such as CT and MRI. Moreover, additional imaging modalities are not always available; the radiation dose burden from CT and the acquisition time and cost of MRI considerably limit the clinical adoption of these techniques.

Two of the most popular PVC algorithms, namely MG and GTM, rely on anatomical/structural information provided by other imaging modalities, such as CT or MRI. Anatomically based methods assume perfect registration and segmentation of multimodal images prior to the application of PVC. In previous studies, the deleterious effect of co-registration errors [33] and segmentation errors [34, 35] on PVC implementation have been investigated and reported, specifically in the context of brain imaging [17, 36–38]. Quarantelli et al. [37] showed that, of all possible sources of error, misregistration errors demonstrated the most substantial impact on the accuracy of PVC in brain PET imaging.

An alternative to these strategies is iterative deconvolution methods [39, 40], which do not require anatomical information or assumptions regarding surrounding structures, tumor size, homogeneity, or background. One drawback of deconvolution-based methods is that they can amplify the high-frequency content of images, thus resulting in increased image noise [41]. As a result, ideal/perfect PVC algorithms appear problematic to achieve [11]. In addition, similar to other PVC methods, deconvolution-based methods still need to incorporate the scanner’s PSF in the reconstruction process [42–44]. As mentioned earlier, accurate characterization of the scanner’s response function could be challenging as it is spatially variable, object-dependent, and can be affected by reconstruction parameters [7]. It has been shown that any PSF mismatch might be critical [28, 45].

Radiomic features analysis evaluates the consistency and robustness of existing patterns in DL-predicted and reference PVC PET images. Considering the relatively poor spatial resolution of clinical PET systems and the importance of PVE in brain PET, conventional radiomic features, such as SUV_max_, SUV_mean_, and total lesion glycolysis (TLG), are expected to be significantly impacted by PVC. Furthermore, high-order features, such as GLZLM which represent small regions/patterns with low gray levels, are essential to evaluate the impact of PVC since PVE can lead to higher bias in small structures. Although our results highlight the importance of radiomic features for the assessment of PVC methods, separate studies are necessary to further understand the relevance of radiomics analysis.

Other assumptions include homogeneity of tracer distribution in a region or tissue component or homogeneous VOI [46, 47]. However, since the VOIs can be very heterogeneous in practice, the homogeneity assumption can introduce uncertainty and bias in parameter estimates [48]. In most voxel-based methods, the correction is valid only for voxels within the target region and requires initial information about the mean or relative mean values in various regions [46]. Region-based methods [42, 49] require manual VOI definition, which suffers from inter- and intra-observer variability. This might potentially lead to different VOI definitions for the same target [50, 51], where the difference in delineation can go up to 15 mm in diameter [52, 53]. In addition, some PVC algorithms require dedicated reconstruction software [42, 54] or extensive parametrization [7, 40, 55].

Research and development efforts are still being spent to tackle the limitations of currently available PVC algorithms. To encourage the clinical community to adopt PVC methods as part of standard processing procedures, more robust and straightforward methods must be developed and made available. It is essential to develop techniques that can be easily integrated, take as few assumptions as possible, and require as little parameter setting as possible.

Similar to other application fields, especially computer vision, DL can be helpful in tackling different problems encountered in PET imaging [56–59]. However, to the best of our knowledge, no DL-based method has been proposed to address the PVE problem in brain PET to date. Application in other body regions, e.g., in clinical oncology, is very sparse, with only a few studies so far [60]. We proposed a method that consists of an end-to-end DL-based pipeline to generate PVC PET images without the need for additional anatomical imaging modality. In addition, it does not depend on any aforementioned underlying assumptions and eliminates the need for prior information, such as VOI size, homogeneity, or regional mean value. We trained and evaluated our proposed model in 83 brain regions defined on a template for various PET neuroimaging radiotracers. The evaluation demonstrated excellent quantitative and qualitative performance. In addition, our method is not affected by the limitations or artifacts present in other imaging modalities or the registration and segmentation inaccuracies commonly existing in alternative methods. One limitation of the current study is that the data were not multi-institutional and were instead collected from a single site. Related to and as a consequence of this, the images were also acquired on the same PET and MRI scanner models. This might affect the generalizability of the model that needs to be addressed in future studies through the use of a more diverse dataset from multiple institutions to further enhance the robustness of the model. Using images acquired on different PET scanners and using different acquisition and reconstruction protocols might improve the robustness and reproducibility of the model, thus leading to better performance. In addition, due to the differing sizes of the datasets for each radiotracer, data augmentation was required. Though this was beneficial in reducing the effect of sample size and increasing the robustness of the model, it may introduce some additional bias. Eliminating the need for additional imaging modalities might be particularly useful in cases where these other modalities are not available or are available but have been acquired in other conditions (e.g., post-operative) or with an important time delay or harbor artifacts that could then be transferred to PET images, as exemplified in Fig. 3. We hope that such end-to-end approaches will facilitate the implementation of PVC in routine clinical setting owing to ease of implementation on different systems. Another limitation of the current study is the absence of an ideal ground truth for the assessment of the proposed PVC technique. The MRI-based PVC method used in this work as a surrogate of the ground truth does not reflect ideal PVC PET images. Despite the advantages of simulations where the ground truth is available for evaluation [60], no simulations/phantoms are capable of perfectly mimicking clinical scenarios. Our model performed better if it was fed with PET images in MNI space. The normalization to MNI space can be automated through simple coding to transfer the images from native space to standard space. This will enable the user to feed the model with images in the native space directly.

PVC has been shown to improve diagnostic accuracy in conditions associated with atrophy and in small brain regions [61]. An added clinical value is also expected in the evaluation of small focal abnormalities, namely the localization of epileptic foci or in the detection of small malignant lesions [62]. Our results demonstrated that the proposed approach provides quantitative accuracy equivalent to alternative approaches without the need for anatomical images.

## Conclusion

This work presents an end-to-end anatomical imaging-free DL-based PVC algorithm to correct for PVE in brain PET imaging. The technique is efficient because it eliminates the need for accurate registration or segmentation or PET scanner response function characterization. In addition, no assumptions regarding VOI size, homogeneity, boundary, or background level are required. The proposed approach fits most situations encountered in the clinical setting and provides sufficient training data. Moreover, it is relatively less sensitive to minor errors that may affect intersubject comparisons and thus is more robust. Given the post-reconstruction nature of the technique, it can be used on existing clinical PET scanners to improve PET’s quantitative accuracy. The qualitative and quantitative performance of the proposed method demonstrated its potential in clinical brain PET studies using various neuroimaging molecular imaging probes. The achieved performance and robustness might make the proposed approach a good candidate for the incorporation of PVC in routine clinical practice.

## Supplementary Information

Below is the link to the electronic supplementary material.

Supplementary file1 (PDF 630 KB)

## Data Availability

Data used in this work are not available owing to privacy/ethical restrictions.
