# Supplementary material for: A cycle-consistent adversarial network for brain PET partial volume correction without prior anatomical information
Source: Eur J Nucl Med Mol Imaging. 2023 Feb 20;50(7):1881–96. doi: 10.1007/s00259-023-06152-0 (PMC10199868; doi:10.1007/s00259-023-06152-0)
Supplement: Supplementary file 1 — Supplementary file1 (PDF 630 KB) [file 259_2023_6152_MOESM1_ESM.pdf]

Supplemental Table 1. Summary of imaging systems and scanning protocols for PET/CT/MR imaging modalities.

	**Imaging systems and scanning protocols**
**PET/CT**	
Scanner model	Biograph mCT scanner (Siemens Healthineers, Erlangen, Germany)
**PET scanner**	
Spatial Resolution in CFOV	4.1 mm FWHM
Sensitivity in CFOV	9.7 ± 0.2 kcps MBq^{-1}
Time-of-Flight (TOF)	527 ps
Voxel size	2.03×2.03×2.2 mm^3
Reconstruction algorithm	Ordinary Poisson Ordered-Subsets Expectation Maximization with TOF (TOF OP-OSEM)
Number of iterations	5
Number of subsets	21
Point Spread Function (PSF)	2 mm FWHM
Gaussian Filtering	
**CT scanner**	SOMATOM® Definition Edge (Siemens Healthineers, Erlangen, Germany)
Tube voltage	120 kVp
Tube current	20 mAs
Rotation time	0.3 s/rotation
Voxel size	0.9 × 0.9 × 2.5 mm^3
**MRI**	
Scanner model	3T MAGNETOM Skyra (Siemens Healthineers, Erlangen, Germany)
Number of head coil channels	64
TR	1930 ms
TI	970 ms
TE	2.3 ms
NEX	1
Flip angle	8°
Voxel size	0.8 × 0.8 × 1 mm^3

Supplemental Table 2. CycleGAN details for generator and discriminator.

**Layer**	**Generators**
**1**	Convolutional-(Filters-32, Kernel size-7, Stride-1)<br>Batch Normalization<br>ReLU
**2**	Convolutional-(Filters-64, Kernel size-3, Stride-2)<br>Batch Normalization<br>ReLU
**3**	Convolutional-(Filters-128, Kernel size-3, Stride-2)<br>Batch Normalization<br>ReLU
**4 - 12**	Residual block-(Filters-128, Kernel size-3, Stride-1)<br>Batch Normalization<br>ReLU
**13**	Convolutional-(Filters-64, Kernel size-3, Stride-0.5)<br>Fractionally strided<br>Batch Normalization<br>ReLU
**14**	Convolutional-(Filters-32, Kernel size-3, Stride-0.5)<br>Fractionally strided<br>Batch Normalization<br>ReLU
**15**	Convolutional-(Filters-3, Kernel size-7, Stride-1)<br>Batch Normalization<br>Tanh
**Layer**	**Discriminators**
**1**	Convolutional-(Filters-64, Kernel size-4, Stride-2)<br>Leaky ReLU with slope 0.2
**2**	Convolutional-(Filters-128, Kernel size-4, Stride-2)<br>InstanceNorm<br>Leaky ReLU with slope 0.2
**3**	Convolutional-(Filters-256, Kernel size-4, Stride-2)<br>InstanceNorm<br>Leaky ReLU with slope 0.2
**4**	Convolutional-(Filters-512, Kernel size-4, Stride-2)<br>InstanceNorm<br>Leaky ReLU with slop 0.2

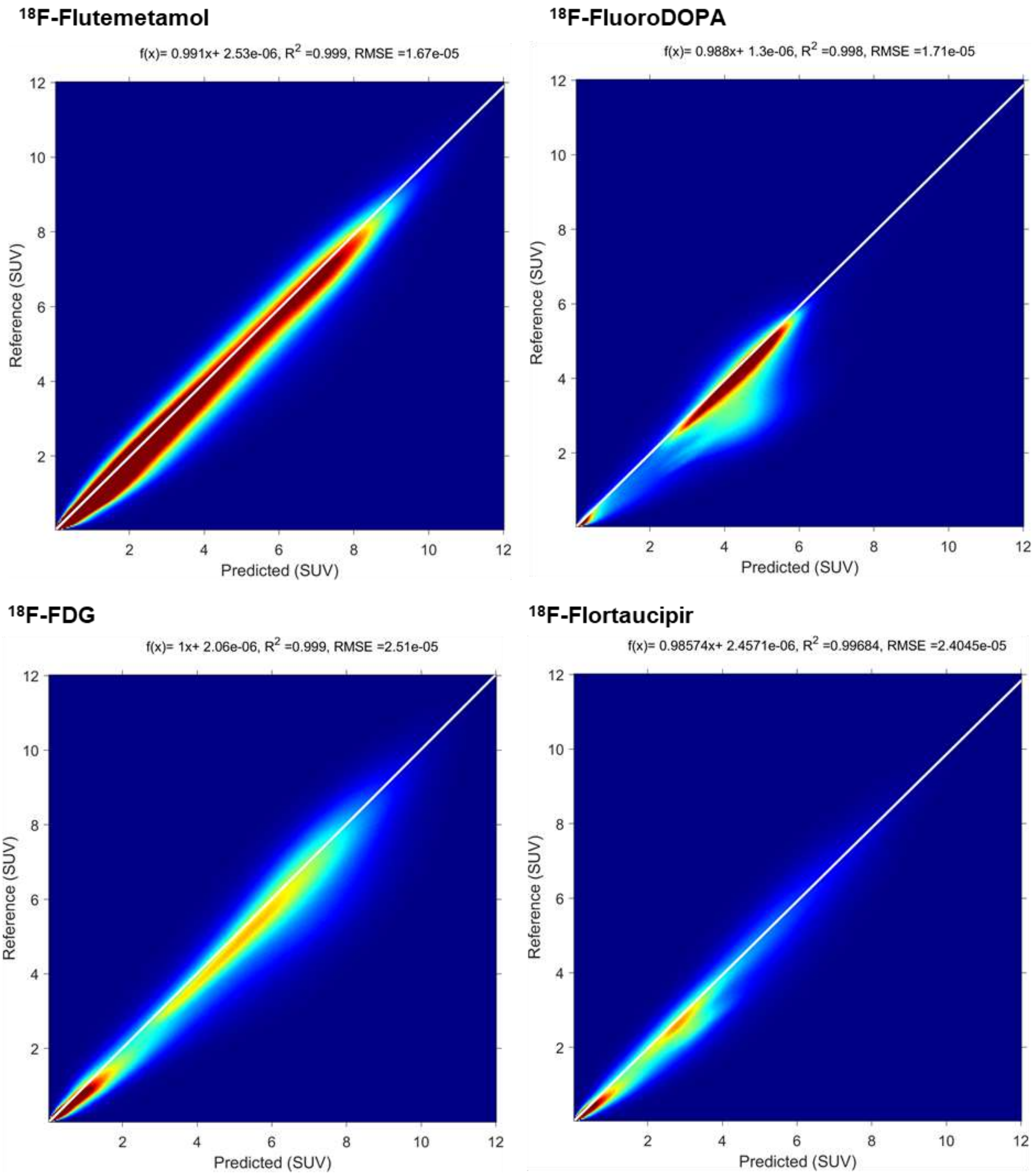

Supplemental Figure 1. Joint voxel-wise SUV histogram analysis between the reference and predicted PVC PET images. For better illustration, the plot was limited to a maximum SUV of 12.

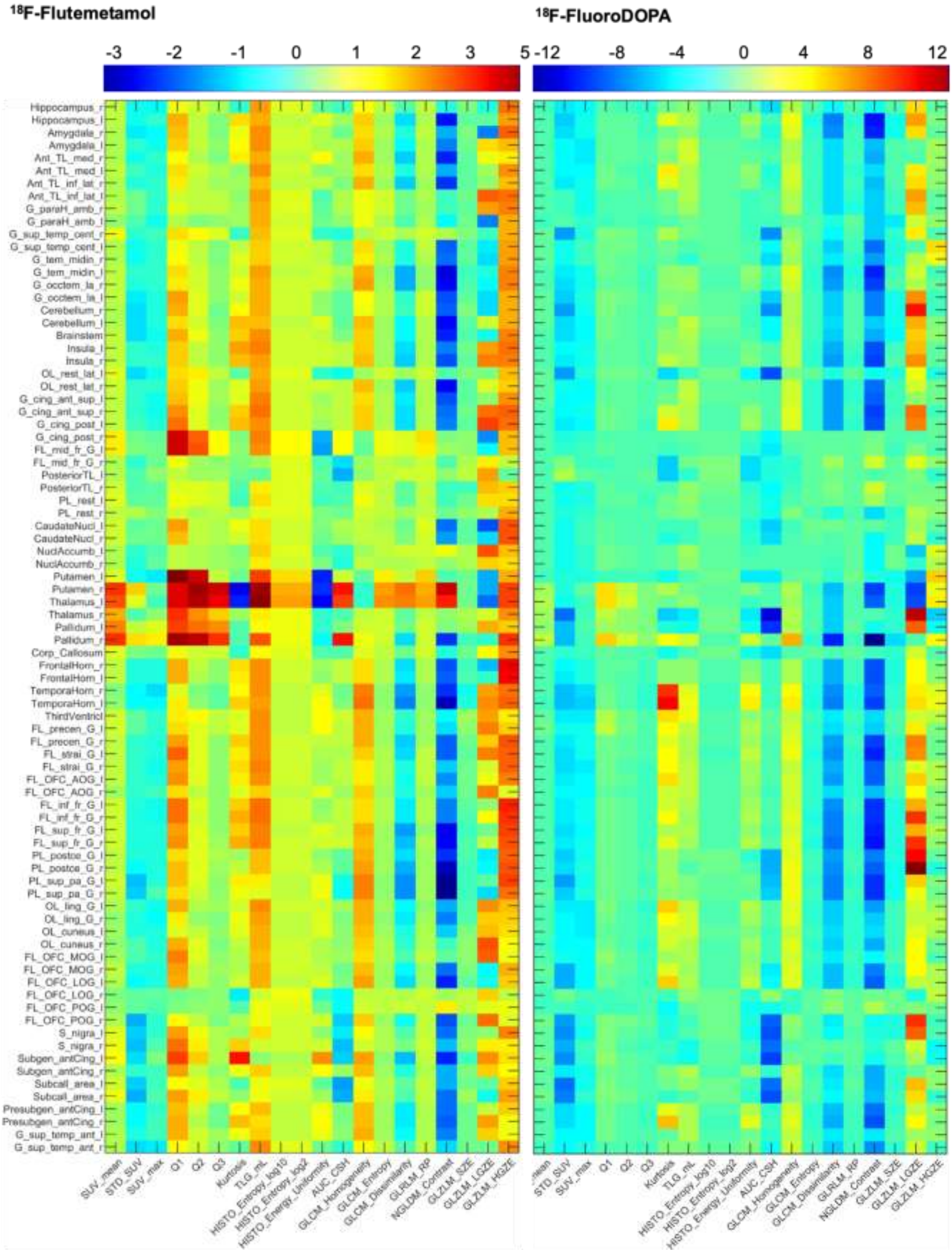

Supplemental Figure 2. Heat map of the relative error of 20 radiomic features calculated across 83 brain regions for the predicted PVC PET images with respect to reference PVC PET images for ^{18}F-Flutemetamol and ^{18}F-FluoroDOPA.

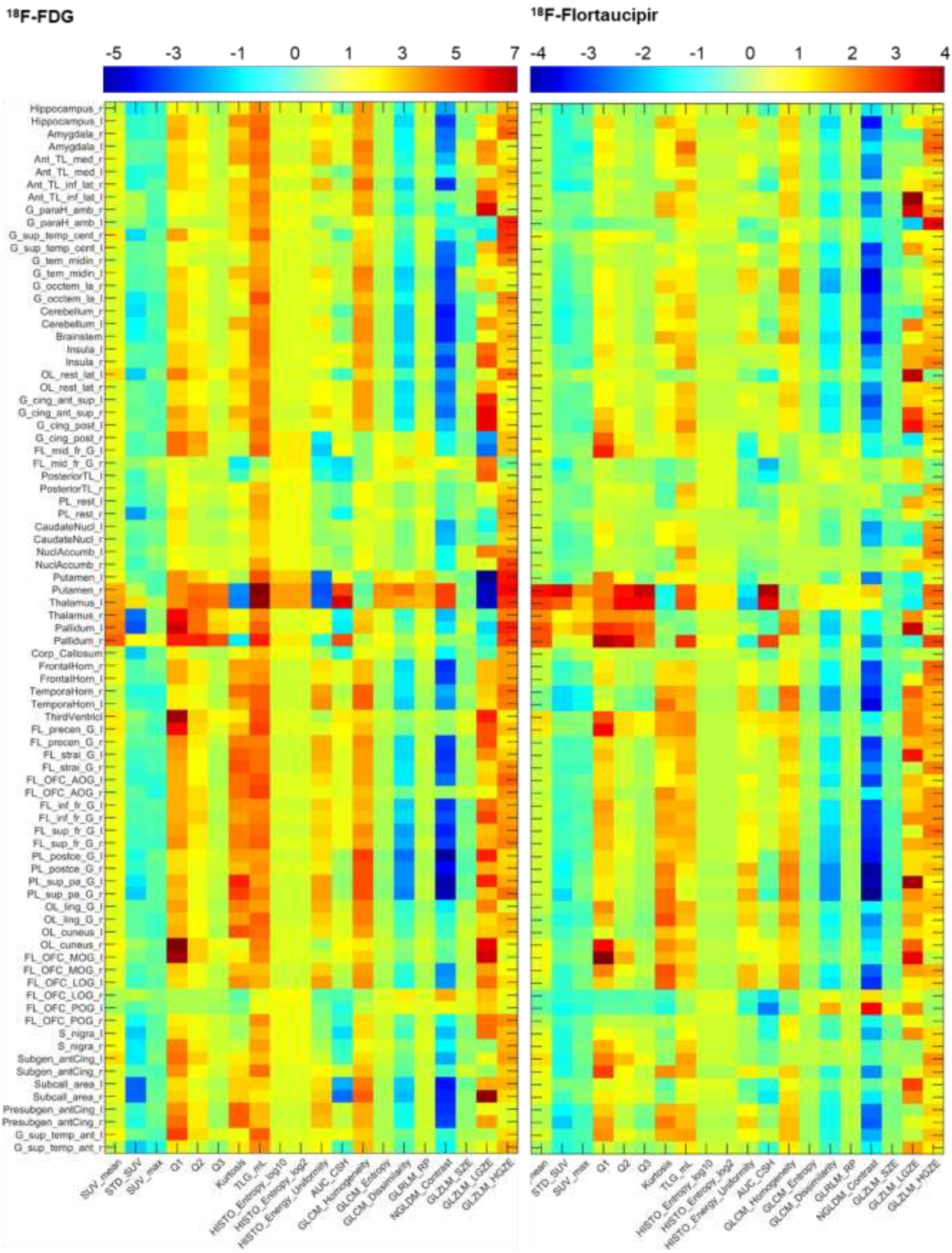

Supplemental Figure 3. Heat map of the relative error of 20 radiomic features calculated across 83 brain regions for the predicted PET PVC images with respect to reference PVC PET images for ^{18}F-FDG and ^{18}F-Flortaucipir.
